# Supplementary material for: Adaptive Laboratory Evolution of Halomonas bluephagenesis Enhances Acetate Tolerance and Utilization to Produce Poly(3-hydroxybutyrate)
Source: Molecules. 2022 May 8;27(9):3022. doi: 10.3390/molecules27093022 (PMC9103988; doi:10.3390/molecules27093022)
Supplement: Supplementary file 1 [file molecules-27-03022-s001.zip › molecules-1696096-supplementary.pdf]

Supporting Information

Table S1. Mutations with gene annotation in B71.

NCBI number	Gene	Position	Description	Amino acid change
WP_009721770.1	Disulfide bond formation protein B	Downstream	540552: A→C 540558: G→A	
WP_009723049.1	FAD-binding oxidoreductase	Exonic	322953: G→T 322956: T→C	185: Gly→Pro 186: Gly→Ser
WP_009723961.1	Methyl-accepting chemotaxis protein	Upstream	264288: C→G 264289: C→A	
WP_009724034.1	BCCT family transporter, partial	Exonic	23041: T→G 23045: C→A	4: Gly→Trp 5: Glu→Ala
WP_009724067.1	Acetoacetyl-CoA reductase	Exonic	59629: G→C 59636: G→T	26: Ala→Ala 29: Gly→Cys
WP_009724268.1	RHS repeat-associated core domain-containing protein	Exonic	272181: A→C	18: Met→Arg
WP_009724802.1	YadA-like family protein, partial	Downstream	52363: G→C 52366: G→T 52375: C→A 52376: C→A 52378: G→T 52382: A→C 52405: G→T	
WP_009724919.1	23S rRNA (cytidine(2498)-2'-O)-methyltransferase RlmM	Upstream	57: A→G	
WP_009725047.1	RHS repeat-associated core domain-containing protein	Exonic	28820: G→C	150: His→Gln
WP_143759813.1	Filamentous hemagglutinin N-terminal domain-containing protein	Exonic	7973: C→T	1128Ala→Val
WP_009725065.1	Hemolysin, partial	Exonic	464: G→T	127: Ala→Asp
WP_009725067.1	Adhesin/hemolysin, partial	Exonic	47: G→A	175: Pro→Leu
WP_009723841.1	5'-nucleotidase C-terminal Domain-containing protein	Exonic	142078: 118 bp In[a]	
WP_009723961.1	Methyl-accepting chemotaxis protein	Upstream	264294: 44 bp In[a]	
WP_009724034.1	BCCT family transporter, partial	Exonic	23050: 30 bp In[a]	
WP_050801109.1	Cell envelope integrity protein TolA	Exonic	175429: 30 bp In[a]	
WP_009724795.1	YadA-like family protein	Exonic	35412: 158 bp Dl[b]	
WP_083817087.1	Histidinol-phosphate transaminase	Downstream	177552: C Dl[b]	
WP_143759813.1	Filamentous hemagglutinin N-terminal domain-containing protein, partial	Exonic	9072: GCC Dl[b]	1494: Ala→no
WP_009722103.1	RHS repeat-associated core domain-containing protein	Exonic	919430:G→T	3: Gln→Thr
WP_009722684.1	Indole-3-glycerol phosphate synthase TrpC	Upstream	616205:G→A 616358:A→G 616372:G→A	
WP_186004630.1	Malate dehydrogenase	Exonic	546801: C→T	14: Val→Ala
WP_009723822.1	PAAR domain-containing protein, partial	Exonic	127706:T→G 127708:G→A	3: Leu→Phe 2: Asp→Gly
WP_009724074.1	SOS response-associated peptidase	Upstream	71786:G→C	
WP_009724730.1	Type VI secretion system tip protein VgrG	Exonic	196008:C→T	485: Lys→Glu
WP_009724776.1	Pyruvate dehydrogenase (acetyl-transferring), E1, homodimeric type	Exonic	3234:G→A 3237:T→C	130: Gly→Gly 129: Lys→Lys
WP_009724787.1	YadA-like family protein, partial	Exonic	15998:C→A 16006:G→A 16172:G→A 16283:A→G	125: Ser→Ser 88: Ser→Ser 33: Val→Lys 30: Ser→Ser
WP_009724794.1	OmpA family protein	Upstream	19715:C→T 19716:T→G 19728:G→A 19731:A→G 19732:G→A 20622:A→G 21072:G→A	
WP_009724795.1	YadA-like family protein	Upstream	35601:G→A 35841:A→G	

			35877:A→G	
			35898:A→G	
			35933:C→T	
			35937:C→G	
			35939:C→T	
			35944:G→C	
			35976:A→T	
WP_009724798.1	Hypothetical protein	Upstream	42545:T→C	
WP_009724802.1	YadA-like family protein, partial	Downstream	51723:G→A	
			51726:A→G	
			51729:T→C	
			51735:C→A	
			51741:G→A	
			51744:G→A	
			59745:G→T	
			59973:G→A	
			59994:G→A	
WP_083817087.1	Histidinol-phosphate transaminase	Downstream	177724:G→T	
WP_009724919.1		Upstream	55:C→A	
			59:G→A	
			65:T→C	
			66:T→A	
			230:A→G	
WP_143759810.1	23S rRNA (cytidine(2498)-2'-O)-methyltransferase RlmM	Exonic	26667:G→A<br>26880:C→G	119: Arg→Arg<br>190: Tyr→Tyr
WP_143759810.1	RHS repeat-associated core domain-containing protein	Downstream	28931:C→G<br>28933:C→T	
WP_009725065.1	Hemolysin, partial	Exonic	582:C→A<br>591:T→C	85: Ala→Thr<br>88: Asp→Tyr
WP_009725067.1	Adhesin/hemolysin, partial	Exonic	214:T→C 371:A→G	66: Ala→Val<br>119: Arg→Arg

[a] In, insertion.
[b] Dl, deletion
